# Fulminant toxic epidermal necrolysis induced by pucotenlimab in a patient with lung adenocarcinoma: a case report

**DOI:** 10.3389/fonc.2026.1790323

**Published:** 2026-06-26

**Authors:** Zhen Zhang, Qiqi Tao, Jing Wang, Jun Lu, Yue Ning

**Affiliations:** Department of Clinical Laboratory, The Quzhou Affiliated Hospital of Wenzhou Medical University, Quzhou People’s Hospital, Quzhou, China

**Keywords:** immune checkpoint inhibitors, irAE, lung adenocarcinoma, pucotenlimab, SJS/TEN

## Abstract

This case report describes a fatal case of toxic epidermal necrolysis (TEN) in a 67-year-old man with stage IIIB lung adenocarcinoma (T_4_N_2_M_0_), which developed 7 days after a single dose of pucotenlimab, a novel programmed death-1 (PD-1) inhibitor. The full course of thoracic radiotherapy was completed prior to immunotherapy initiation, and no persistent hypersensitivity reactions were documented despite concomitant antibiotic administration throughout this period. The patient presented with scattered erythematous papules that rapidly progressed to extensive epidermal detachment involving approximately 80% of the total body surface area (BSA). A baseline SCORTEN score of 6 calculated within 24 hours of readmission (**Table 1**) indicated a predicted mortality >90%, and the Naranjo Scale yielded a score of 6 (**Table 2**), confirming a probable causal association between TEN and pucotenlimab. Acute-phase immunological profiling revealed markedly reduced PD-1 expression on circulating T cells and significantly elevated interleukin-6 (IL-6), providing exploratory evidence for aberrant T-cell activation as a potential pathogenic mechanism. Despite intensive multidisciplinary management with systemic corticosteroids, intravenous immunoglobulin (IVIG) and supportive care, the patient’s condition progressively deteriorated. After written informed consent for withdrawal of life-sustaining treatment was obtained from the patient’s legal next of kin, the patient was discharged on July 31 and succumbed to his illness shortly thereafter. This case highlights the fatal risk of rare severe cutaneous immune-related adverse events (irAEs) associated with PD-1 inhibitors. It also emphasizes the importance of early recognition, prompt discontinuation of further PD-1 inhibitor administration, and timely combination therapy with corticosteroids and etanercept to improve outcomes in high-risk patients.

## Introduction

Stevens–Johnson syndrome (SJS) and toxic epidermal necrolysis (TEN) are rare but life-threatening mucocutaneous hypersensitivity reactions characterized by extensive keratinocyte apoptosis and dermo–epidermal separation, leading to profound blistering and mucosal involvement (1). Historically, these conditions have been primarily triggered by medications such as nonsteroidal anti-inflammatory drugs (NSAIDs), anticonvulsants, and β-lactam antibiotics (2).

**Table 1:** SCORTEN assessment upon admission.

Variable	Score 0 (normal)	Score 1 (abnormal)	Patient findings	Patient’s score
Age	< 40 years	≥ 40 years	67 years	1
Associated malignancy	No	Yes	Diagnosed with stage IIIB lung adenocarcinoma	1
Heart rate	< 120bpm	≥120bpm	126 bpm	1
Serum BUN	≤10 mmol/L	> 10 mmol/L	11.45 mmol/L	1
Epidermal detachment	< 10% BSA	≥10% BSA	23% BSA (initial)	1
Serum bicarbonate	≥20 mmol/L	< 20 mmol/L	19.3 mmol/L	1
Serum glucose	≤ 250 mg/dL	> 250 mg/dL	232.2 mg/dL	0
Total SCORTEN	—	—	—	6

SCORTEN is calculated by summing 1 point for each positive factor. Predicted mortality rates: 0–1 (3.2%), 2 (12.1%), 3 (35.3%), 4 (58.3%), and ≥5 (≥90%).

bpm, beats per minute; BSA, body surface area; BUN, blood urea nitrogen.

**Table 2:** Naranjo adverse drug reaction probability scale for pucotenlimab.

No.	Assessment question	Yes	No	Unknown	Score	Rationale
1	Are there previous conclusive reports on this reaction?	+1	0	0	1	Cutaneous adverse events, including SJS/TEN, are documented for PD-1 inhibitors in literature.
2	Did the adverse event appear after the suspected drug was administered?	+2	-1	0	2	Cutaneous symptoms manifested on day 6 following the initial infusion of Pucotenlimab.
3	Did the reaction improve when the drug was discontinued or a specific antagonist was given?	+1	0	0	0	Clinical improvement was confounded by concurrent high-dose corticosteroids and IVIG therapy.
4	Did the reaction reappear when the drug was readministered?	+2	-1	0	0	Re-challenge was clinically contraindicated due to the severity of the reaction.
5	Are there alternative causes (other than the drug) that could have caused the reaction?	-1	+2	0	2	Concomitant antibiotics were administered prior to TEN onset without hypersensitivity; temporal pattern favored Pucotenlimab.
6	Did the reaction reappear when a placebo was given?	-1	+1	0	0	Placebo challenge was not performed.
7	Was the drug detected in the blood (or other fluids) in concentrations known to be toxic?	+1	0	0	0	Serum drug concentration monitoring was not performed.
8	Was the reaction more severe when the dose was increased, or less severe when the dose was decreased?	+1	0	0	0	The patient received only a single therapeutic dose of Pucotenlimab.
9	Did the patient have a similar reaction to the same or similar drugs in any previous exposure?	+1	0	0	0	The patient was naive to immune checkpoint inhibitors (ICIs).
10	Was the adverse event confirmed by any objective evidence?	+1	0	0	1	Confirmed by characteristic clinical findings of TEN, including >80% BSA detachment and positive Nikolsky sign.
	Total Score				6	Interpretation: Probable ADR

ADR Probability Scale Interpretation.

Definite (≥9): The reaction followed a reasonable temporal sequence, followed a recognized response to the drug, was confirmed by withdrawal and re-exposure.

Probable (5–8): The reaction followed a reasonable temporal sequence, followed a recognized response, and could not be reasonably explained by the patient's clinical state.

Possible (1–4): The reaction followed a temporal sequence but could also be explained by the patient's disease or other therapies.

Doubtful ( ≤ 0): The reaction was likely related to factors other than the drug.

However, the rapid expansion of immune checkpoint inhibitors (ICIs) in oncology has introduced a novel spectrum of immune-related adverse events (irAEs). ICIs, including pucotenlimab, ipilimumab, and nivolumab, exert antitumor effects by blocking inhibitory immune checkpoints such as programmed death-1 (PD-1), programmed death ligand-1 (PD-L1), and CTLA-4, thereby enhancing T-cell-mediated immune surveillance (3). Pucotenlimab is a novel humanized IgG4 monoclonal antibody targeting PD-1 that has been approved in China for the treatment of selected solid tumors (4). As of April 2026, it has not been approved in other countries or regions.

While cutaneous irAEs such as maculopapular rash and pruritus are relatively common, severe cutaneous adverse reactions (SCARs), including SJS and TEN, remain rare but potentially fatal (5). Notably, to our knowledge, as of April 2026, only a handful of pucotenlimab-associated TEN cases have been reported in PubMed and Embase databases, with no published studies providing detailed acute-phase immune phenotyping and cytokine profiling to elucidate the underlying immunopathological mechanism. The clinical characteristics, pathogenesis, and optimal management of this rare fatal adverse event therefore remain poorly understood.

Here, we report a case of fulminant TEN occurring after pucotenlimab therapy in a patient with lung adenocarcinoma. We systematically evaluated the causal association using established clinical severity and causality assessment tools, including the SCORTEN score and Naranjo Scale, and performed acute-phase immunological profiling to elucidate the potential immunopathological drivers of this severe reaction. This report aims to supplement the scarce post-marketing safety data of pucotenlimab, provide novel exploratory immunopathological evidence for ICI-related TEN, and guide clinical decision-making for the early recognition and standardized management of this fatal adverse event.

## Case report

This study was conducted in compliance with the postulates of Declaration of Helsinki and approved by the Human Ethics Committee of the Quzhou Hospital of Wenzhou Medical University (2024–173). The subject of this case report and his family members have provided their written informed consent for the publication of his case, including the disclosure of relevant images.

A 67-year-old man (73.6 kg, 170 cm, body mass index (BMI) 25.47 kg/m²) with no significant past medical history, including hypertension, diabetes, or coronary artery disease, was admitted to our hospital on June 3, 2025, because of hemoptysis for 3 days and detection of a lung mass 2 days earlier. He reported no known drug allergies or family history of hereditary disease. He had a 30-pack-year smoking history and had quit 5 years previously.

Chest computed tomography (CT) performed at another hospital on June 2 revealed a mass in the lingular segment of the left upper lobe, with mediastinal and hilar lymph node metastases. On June 5, positron emission tomography–CT (PET-CT) at our hospital showed a hypermetabolic mass in the left upper lobe, highly suggestive of lung cancer, with local bronchial truncation and possible pulmonary artery invasion. Increased fluorodeoxyglucose (FDG) uptake was also observed in mediastinal lymph nodes (stations 4R/L, 7, 10L, and 11L), indicating probable metastatic involvement.

On June 6, sedated bronchoscopy with biopsy confirmed lung adenocarcinoma. Immunohistochemical analysis showed TTF-1 (+), CK7 (+), Napsin A (+, approximately 30%), Ki-67 (+), SP-A (+), SMARCB1 (+), SMARCA4/Brg1 (+), and weak CDX-2 positivity, while P40, CK20, and CK5/6 were negative. PD-L1 testing repeated on June 17 showed a tumor proportion score (TPS) of 75% and a combined positive score (CPS) of 76. The patient was diagnosed with stage IIIB non-small cell lung cancer (lung adenocarcinoma, T_4_N_2_M_0_).

After admission, empirical anti-infective therapy was sequentially initiated: cefoperazone/sulbactam (3 g every 12h) was administered from June 13 to June 25, 2025, followed by meropenem (1 g every 8h) from June 25 to July 3, 2025. No persistent hypersensitivity reactions to the above β-lactam antibiotics were observed during treatment, except for transient localized abdominal allergic dermatitis on June 14, which resolved completely after symptomatic treatment. Radiotherapy was delivered from June 16 to June 20, 2025, with a prescribed dose of 25 Gy in 5 fractions over 7 days to the planning gross tumor volume (PGTV) and regional lymph nodes (PGTVnd), and the full course of radiotherapy was completed before immunotherapy initiation. On July 3, the patient received a single intravenous dose of pucotenlimab (200 mg), according to the standard approved dosing regimen in China. Following completion of radiotherapy, the patient demonstrated significant symptomatic improvement, with markedly relieved chest tightness and no cough, sputum production, chest pain, abdominal distension, or diarrhea. Vital signs were stable, with a Karnofsky Performance Status (KPS) score of 80, and physical examination revealed no significant abnormalities. As the patient met routine discharge criteria and strongly requested discharge for personal reasons, he was discharged home on July 4 for continued convalescence.

**Figure 1:**
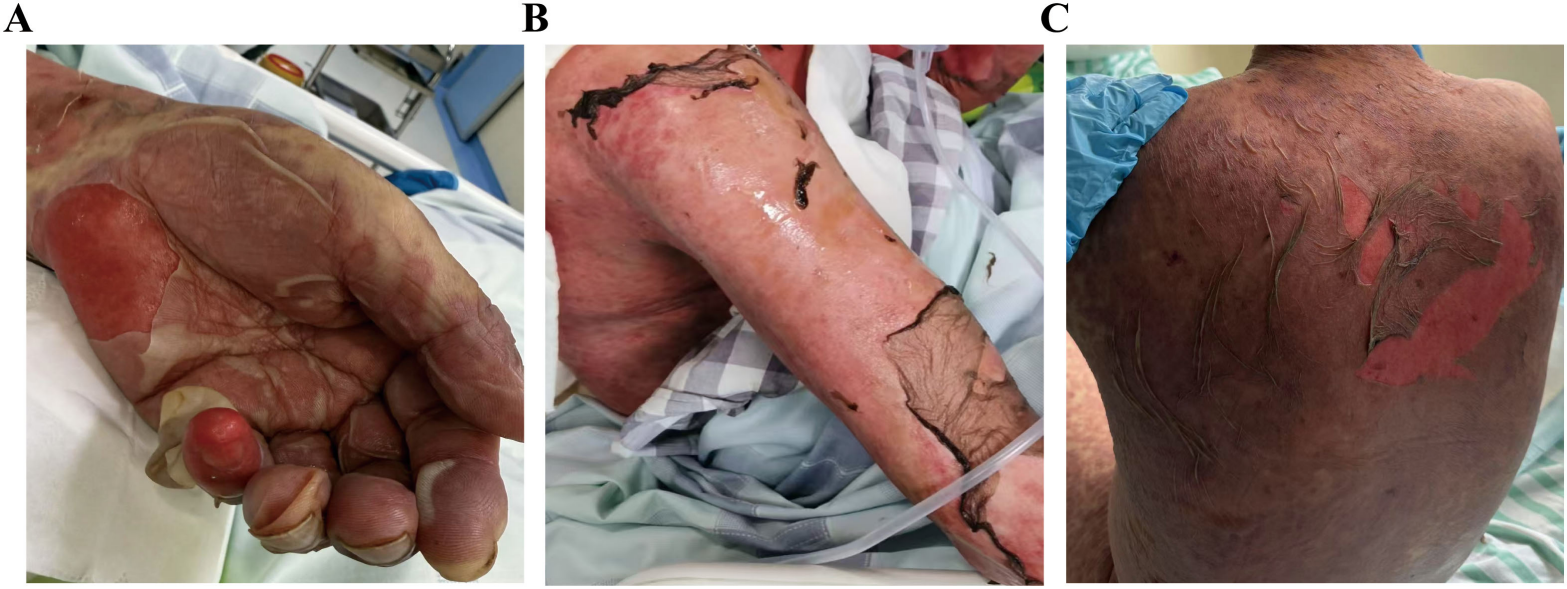
Representative acute cutaneous manifestations on July 12 Generalized erythema and extensive epidermal detachment were observed, with a positive Nikolsky’s sign, consistent with typical toxic epidermal necrolysis lesions. **(A)** Close-up of the palm, showing large sheets of epidermal denudation with residual flaccid necrotic epidermis overlying a bright erythematous erosive base. **(B)** Lesions spanning the upper arm to forearm, presenting with widespread epidermal detachment, moist erythematous wounds, and dark necrotic epidermis along the desquamation margins. **(C)** Lesion on the upper back, with extensive loose and detached epidermis exposing underlying raw erythematous erosive surfaces.

On the sixth day after discharge (July 10), the patient presented with a generalized pruritic erythematous rash, which rapidly deteriorated and evolved into extensive epidermal sloughing characteristic of TEN. He was consequently readmitted on July 12 for severe mucocutaneous toxicity (**Figure 1**). Physical examination revealed extensive mucocutaneous involvement consistent with acute toxic epidermal necrolysis (TEN), including widespread epidermal necrosis and detachment. The necrotic epidermis appeared brownish and semitransparent, with complete epidermal loss exposing a bright red, moist dermal surface in multiple areas. Nikolsky’s sign was strongly positive. Epidermal detachment initially involved 23% of the BSA and rapidly progressed to 80% by July 21. A baseline SCORTEN score of 6 was calculated within 24 hours of readmission, with each abnormal item and corresponding patient findings as follows: age ≥40 years (67 years, 1 point), presence of malignancy (stage IIIB lung adenocarcinoma, 1 point), heart rate ≥120 bpm (126 bpm, 1 point), blood urea nitrogen >10 mmol/L (11.45 mmol/L, 1 point), initial epidermal detachment ≥10% BSA (23%, 1 point), and serum bicarbonate <20 mmol/L (19.3 mmol/L, 1 point). This score indicated a predicted in-hospital mortality rate of >90% (**Table 1**).

Laboratory tests on readmission showed a white blood cell count of 10.9 × 10^9/L, neutrophil count of 8.18 × 10^9/L, and high-sensitivity C-reactive protein (hs-CRP) level of 190.62 mg/L, consistent with severe systemic inflammation. Serum biochemistry showed acute kidney injury (AKI) (creatinine 178.0 μmol/L) and hyperkalemia (potassium 5.74 mmol/L). D-dimer was elevated at 8.39 mg/L, suggesting coagulation activation.

On July 13, the patient was transferred to the intensive care unit for electrocardiographic monitoring, mechanical ventilation via tracheal intubation, and protective isolation. Systemic immunosuppressive therapy was initiated with intravenous methylprednisolone (80 mg/day) and intravenous immunoglobulin (IVIG; 20 g/day for 3 consecutive days; cumulative dose approximately 0.8 g/kg). A multidisciplinary team consisting of dermatologists, intensivists, and ophthalmologists was convened to manage fluid resuscitation, prevent mucosal sequelae, and optimize topical burn ointment treatment. Anti-infective therapy included intravenous meropenem (1.0 g every 8 h) and linezolid (0.6 g every 12 h), together with comprehensive nutritional support and hepatoprotective treatment.

**Figure 2:**
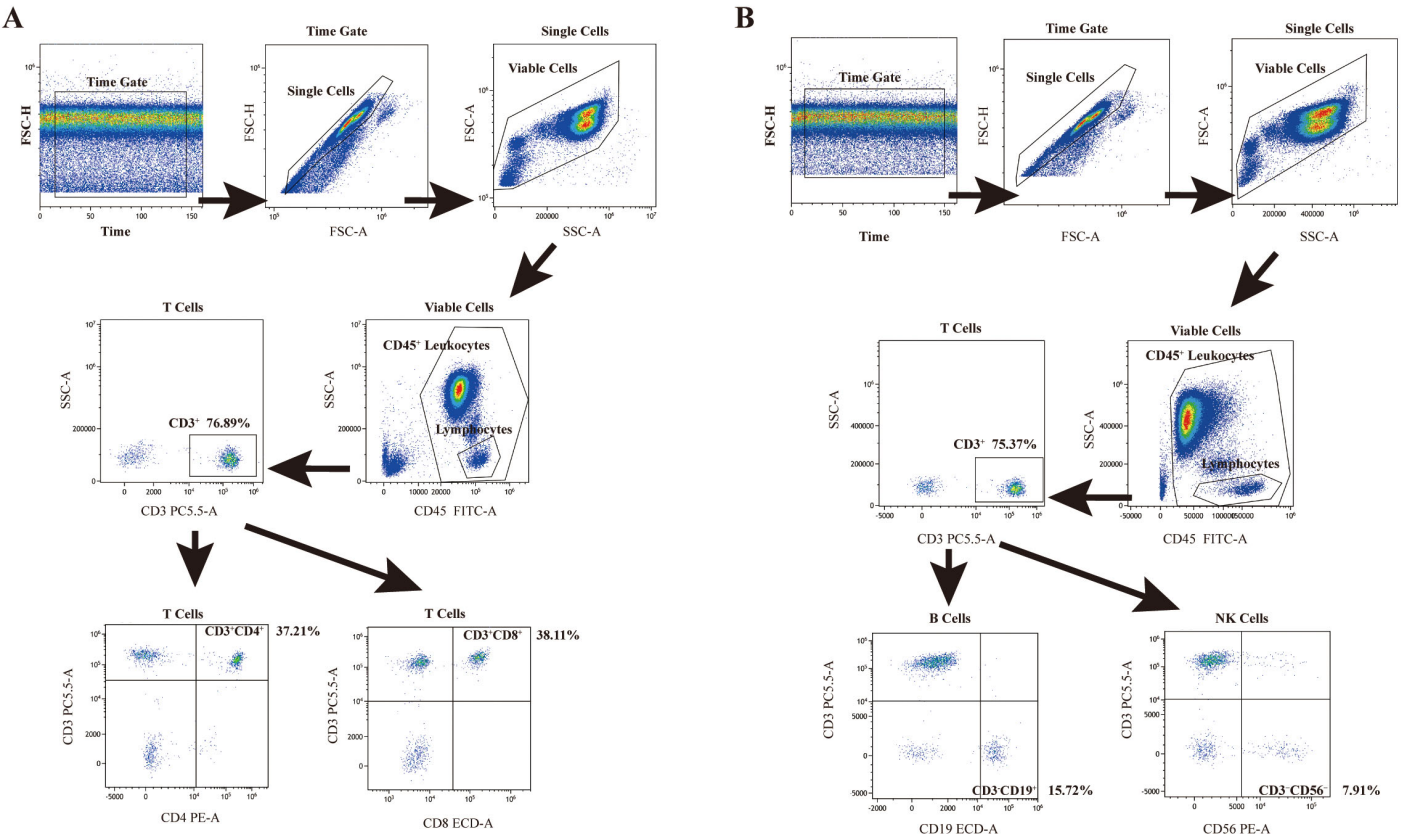
Flow cytometric analysis of peripheral blood immune cell subsets during the acute phase of TEN. **(A)** Gating strategy for CD3^+^T cells and their CD4^+^/CD8^+^ subsets. Events were sequentially gated for stable acquisition, singlets, viable cells, CD45^+^leukocytes, and lymphocytes. Total CD3^+^T cells were further divided into CD3^+^CD4^+^and CD3^+^CD8^+^subsets. **(B)** Gating strategy for B cells, NK cells, and NKT cells. Upstream gating was identical to **(A)**. CD3^−^lymphocytes were identified as B cells (CD3^−^CD19^+^) and NK cells (CD3^−^CD56^+^), while CD3^+^CD56^+^ cells were defined as NKT cells.

**Figure 3:**
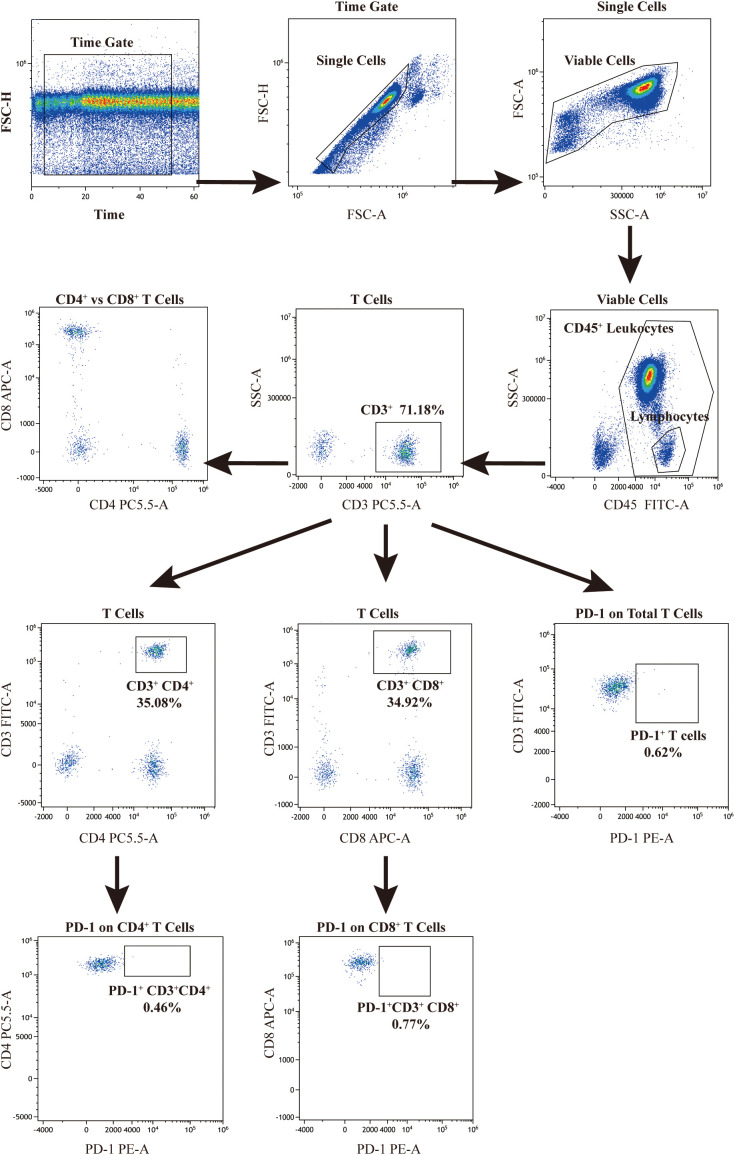
Flow cytometric assessment of PD-1 expression on circulating T cells during the acute phase of TEN. Representative flow cytometry plots demonstrating PD-1 expression on CD3^+^ total T cells, CD3^+^CD4^+^ helper T cells, and CD3^+^CD8^+^ cytotoxic T cells. The identical upstream gating strategy described in **Figure 2** was applied. PD-1 expression was analyzed separately on total CD3^+^ T cells, purified CD3^+^CD4^+^ T cells, and purified CD3^+^CD8^+^ T cells.

On July 14, within 48 hours of intensive care unit (ICU) admission (corresponding to the acute progressive phase of TEN), peripheral venous blood samples were collected for comprehensive immune profiling and cytokine quantification. Immune cell phenotyping was performed via flow cytometry, and serum cytokine levels were measured using a cytometric bead array (CBA) assay (**Figure 2**). No baseline immune profiling data were available for comparison, as exploratory immunological blood samples were not obtained prior to pucotenlimab administration. Flow cytometric analysis revealed that CD3^+^ T cells constituted 76.89% of total lymphocytes, comprising 37.21% CD3^+^CD4^+^ helper T cells and 38.11% CD3^+^CD8^+^ cytotoxic T cells, yielding a CD4^+^/CD8^+^ ratio of 0.97. Natural killer (NK) cells and B cells accounted for 7.91% and 15.72% of total lymphocytes, respectively. CD3^+^CD56^+^ NKT cells were present at a low frequency, and no significant phenotypic abnormalities were detected in any lymphocyte subset. Notably, PD-1 expression on circulating T cells was markedly reduced compared to our laboratory’s healthy adult reference ranges. Specifically, PD-1 was expressed on 0.62% of total CD3^+^ T cells, 0.46% of CD3^+^CD4^+^ helper T cells, and 0.77% of CD3^+^CD8^+^ cytotoxic T cells (reference ranges: CD4^+^ PD-1^+^: 1.2%-6.8%, CD8^+^ PD-1^+^: 2.1%-9.5%) (**Figure 3**). Cytokine profiling demonstrated a dramatic elevation in interleukin-6 (IL-6) at 223.94 pg/mL (reference range: 0-5.9 pg/mL). Tumor necrosis factor-α (TNF-α) was within the normal range at 2.58 pg/mL (reference range: 0-8.1 pg/mL), and levels of IL-2, IFN-γ, IL-10, IL-17, and IL-4 remained unremarkable. Although hs-CRP (66.76 mg/L) and serum creatinine (128.2 μmol/L) had decreased slightly by July 14, the patient’s clinical course remained unstable (**Table 3**).

**Table 3:** Comparison of inspection indicators.

Parameter	First examination	Last examination	Unit
IL-2	0.57	1.81	pg/mL
IL-6	60.32	223.94	pg/mL
IL-10	4.24	25.90	pg/mL
IFN-γ	3.67	6.71	pg/mL
IL-17	4.56	3.98	pg/mL
IL-4	0.93	2.15	pg/mL
TNF-α	2.25	2.58	%
CD19+%	0.3	15.72	%
CD3+CD4+%	17.7	37.21	%
CD3+CD8+%	51.8	38.11	%
CD3-CD56+%	21.8	7.91	%
CD4+/CD8+	0.34	0.98	
WBC	7.8	4.4	×10^9^/L
RBC	5.06	2.37	×10¹²/L
HGB	143	63	g/L
PLT	267	66	×10^9^/L
LY	1.54	0.08	×10^9^/L
LY%	19.7	1.8	%
hs-CRP	13.06	368.65	mg/L
PCT	0.26	0.10	ng/mL
CREA	103.2	165.2	μmol/L
CHE	6373	2563	U/L
DBIL	0.9	7.3	μmol/L
K	3.74	5.87	mmol/L
P	0.99	1.84	mmol/L
CK	54.7	296.7	U/L

WBC, white blood cell count; RBC, red blood cell; HGB, haemoglobin; LY%, lymphocyte; PLT, platelet count/blood platelet count; PCT, plateletocri; CREA, creatinine; CK, creatine kinase; hs-CRP, hypersensitive C-reactive protein; K, kalium; DBIL, direct bilirubin; CHE, Cholinesterase; P, phosphate; IL-2, Interleukin-2; IL-6, Interleukin-6; IL-10, Interleukin-10; IL-17, Interleukin-17; IL-4, Interleukin-4; IFN-γ, Interferon-γ; TNF-α, Tumor necrosis factor-α.

**Figure 4:**
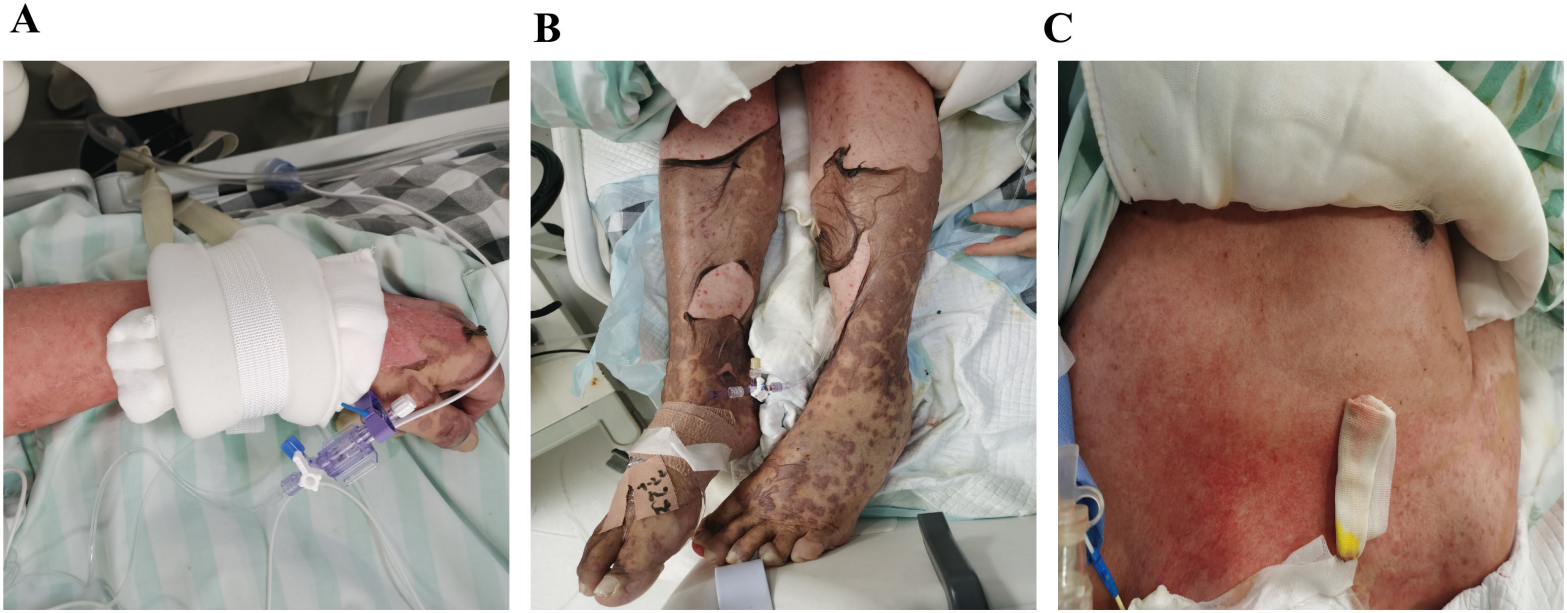
Skin manifestations during the recovery phase on July 22 The pre-existing erythematous and exfoliative lesions had gradually crusted over with progressive re-epithelialization, and no newly formed blisters were observed. **(A)** Residual diffuse erythema on the forearm, with flaccid necrotic epidermis remaining on the hand. **(B)** Bilateral lower legs and feet showing widespread residual dark necrotic epidermis with mottled pigmentation, with re-epithelialized surfaces exposed beneath areas of spontaneous desquamation. **(C)** Abdominal skin with faded erythema and nearly complete re-epithelialization, accompanied by scattered post-inflammatory pigmentary changes.

By July 22, the cutaneous lesions had entered a recovery phase, with widespread brown crust formation, a negative Nikolsky’s sign, and newly formed epithelium without active blistering (**Figure 4**). However, in a tragic paradox, the systemic infection concurrently worsened, progressing to fungal sepsis and septic shock, as confirmed by Pulse Contour Cardiac Output (PiCCO) monitoring, which showed high cardiac output and low systemic vascular resistance. Hematologic parameters deteriorated markedly (hemoglobin 69 g/L, platelets 78 × 10^9/L), and AKI required continuous renal replacement therapy (CRRT). The antimicrobial regimen was escalated to include daptomycin, meropenem, and prophylactic fluconazole.

**Figure 5:**
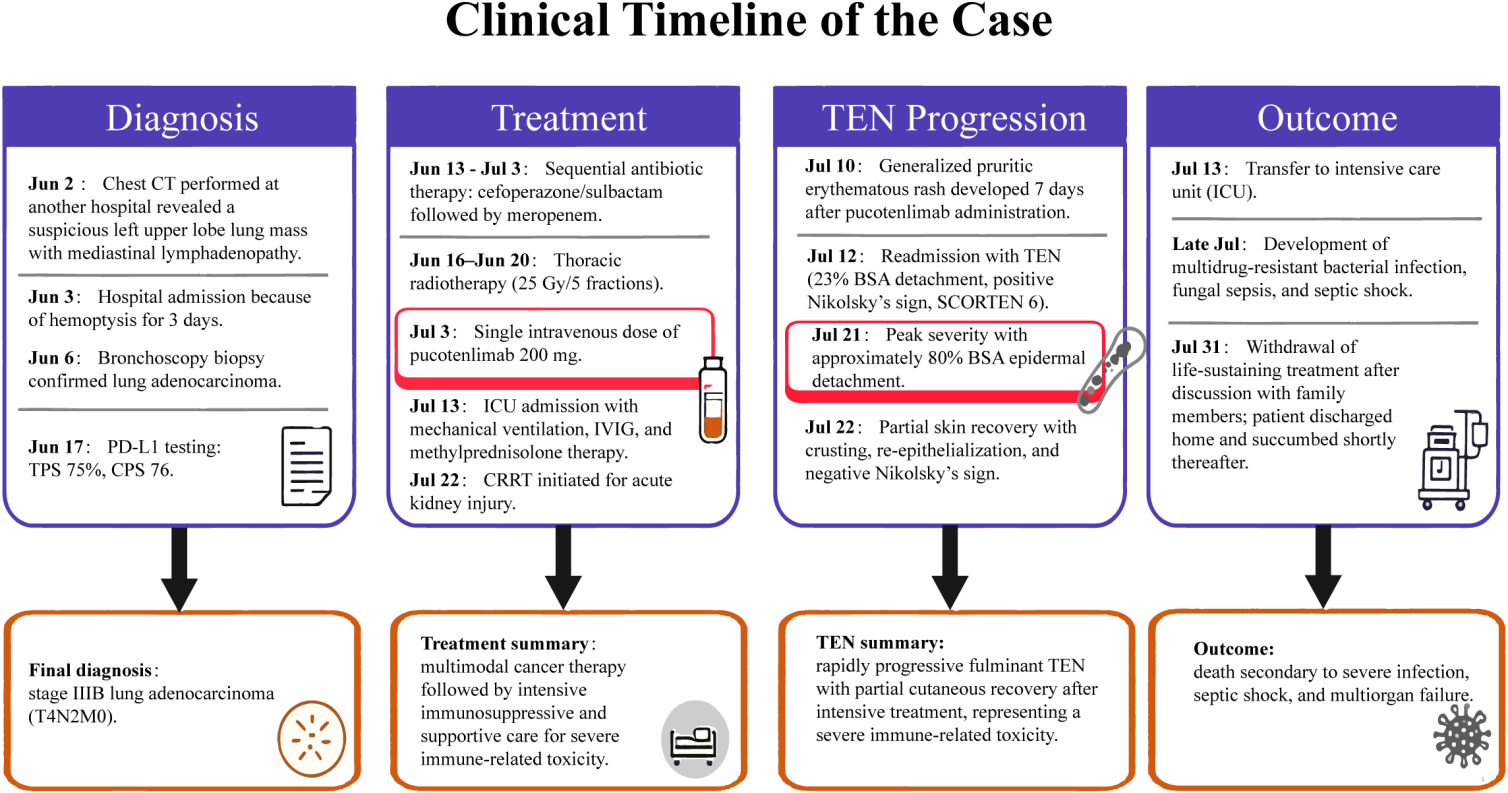
Clinical timeline of the patient’s entire clinical course.

In the final phase of treatment, cultures confirmed multidrug-resistant Acinetobacter baumannii from the catheter and Candida tropicalis from the blood. Despite maximal supportive care, including vasoactive agents to maintain hemodynamic stability and continuous renal replacement therapy (CRRT) for severe multiorgan failure, the patient’s condition continued to deteriorate irreversibly. On July 31, after full communication with the patient’s family and obtaining written informed consent for treatment withdrawal, active treatment was discontinued due to the patient’s critical condition and extremely poor prognosis, and the patient was discharged home at the family’s request. (**Figure 5**).

## Discussion

Immune checkpoint inhibitor (ICI)-related adverse events (irAEs) represent a spectrum of immune-mediated toxicities resulting from nonspecific activation of the immune system, with the skin being among the most frequently involved organs (6, 7). Toxic epidermal necrolysis (TEN) is a rare but life-threatening mucocutaneous reaction characterized by extensive epidermal detachment and high mortality.

In the present case, TEN developed 7 days after a single dose of pucotenlimab, a programmed death-1 (PD-1) inhibitor, with involvement of approximately 80% of the body surface area. The temporal relationship, together with a Naranjo score of 6, supports a probable association between pucotenlimab and TEN (8).

Notably, we consider prior antibiotic therapy to be an unlikely contributor, while thoracic radiotherapy remains a potential trigger, based on the following evidence: First, for β-lactam antibiotics, cefoperazone-sulbactam was discontinued 15 days before TEN onset, and meropenem was discontinued 7 days before rash onset. This time window is completely inconsistent with the classic pathogenesis of β-lactam-induced TEN, which typically has a sensitization incubation period of 7–14 days for initial exposure, and onset rarely exceeds 1 week after drug discontinuation. Furthermore, no persistent or progressive mucocutaneous hypersensitivity reactions were observed during antibiotic administration, further ruling out the causal role of β-lactams. Second, for thoracic radiotherapy, the full course of 25 Gy in 5 fractions was completed 20 days before TEN onset, far exceeding the conventional onset window of radiation-induced skin injury (during radiotherapy to 1–2 weeks after completion). More importantly, the rash was generalized throughout the body, mainly involving the trunk and bilateral thighs, with no preferential involvement within the thoracic irradiation field, which is completely inconsistent with the clinical characteristics of radiation dermatitis. Generalized TEN induced by conventional fractionated thoracic radiotherapy is extremely rare in clinical practice, thus radiotherapy is unlikely to have been the primary trigger. The acute onset, extensive epidermal detachment, and strongly positive Nikolsky’s sign, in the absence of marked eosinophilia or typical target lesions, also made alternative diagnoses including drug reaction with eosinophilia and systemic symptoms (DRESS) and erythema multiforme major less likely. Skin biopsy was not performed due to the patient’s critical condition and the high risk of procedure-related bleeding and infection.

To further explore the underlying immunological mechanisms, peripheral immune profiling was conducted. Flow cytometric analysis demonstrated a relatively preserved distribution of T-cell subsets, with a balanced CD4^+^/CD8^+^ ratio of 0.97 and no apparent abnormalities in B-cell or natural killer cell populations. Notably, PD-1 expression on both CD4^+^ and CD8^+^ T cells was markedly reduced. In chronic malignancy, sustained antigen exposure is typically associated with T-cell exhaustion and increased PD-1 expression (9). In contrast, the extremely low PD-1 expression on both CD4^+^ and CD8^+^ T cells observed in this case (0.46% and 0.77%, respectively, far below the normal reference range) may suggest that the patient’s T cells were not in a profoundly exhausted state, but rather in a quiescent state with preserved proliferative and activation potential. Following PD-1 blockade, such cells may be more susceptible to dysregulated activation, potentially contributing to off-target immune injury directed against keratinocytes through cytotoxic pathways involving perforin and granzyme B (10–12). No baseline immunological profiling data were obtained before pucotenlimab exposure, as pre-treatment peripheral blood samples were not reserved for flow cytometric research purposes. Thus, the immunological findings reported herein represent acute-phase changes of TEN, and the mechanistic interpretation should be considered preliminary.

Consistent with this hypothesis, cytokine analysis revealed a markedly elevated IL-6 level and a moderate increase in TNF-α, while other cytokines remained relatively low. IL-6 is a key mediator of inflammatory amplification in irAEs and has been implicated in the pathogenesis of severe cutaneous adverse reactions. The observed cytokine profile suggests the presence of a systemic hyperinflammatory state. Correspondingly, the patient developed progressive hematologic abnormalities, hepatic dysfunction, acute kidney injury (AKI), and electrolyte disturbances, indicating the presence of multiorgan involvement. These findings support the concept that TEN represents a systemic inflammatory syndrome rather than an isolated cutaneous disorder.

From a clinical perspective, the prognosis of ICI-associated TEN is poor, particularly when complicated by infection, shock, or multiorgan failure. Management strategies include prompt discontinuation of the suspected agent, intensive supportive care, and early initiation of immunomodulatory therapy. Emerging evidence supports the use of combination therapy with corticosteroids and tumor necrosis factor-α inhibitors, such as etanercept, which may improve clinical outcomes compared with corticosteroids alone (13). In this case, the patient did not receive early TNF-α inhibitor therapy due to the high infection risk and renal insufficiency at admission, which may have contributed to the irreversible disease progression and fatal outcome. These observations suggest that early combined immunomodulatory therapy, including consideration of TNF-αinhibitors such as etanercept, may warrant further investigation in high-risk ICI-related TEN. Treatment should be individualized based on disease severity, as assessed by tools such as the SCORTEN score, and careful attention should be paid to infection control and organ support (14).

Several limitations should be acknowledged. First, immune profiling was performed at a single time point during the acute phase of TEN, with no baseline measurements obtained before pucotenlimab administration for comparison, which limits the definitive interpretation of dynamic immune changes. The reduced PD-1 expression may partially reflect dynamic immune redistribution during severe systemic inflammation. In addition, the absence of histopathological confirmation via skin biopsy limits direct assessment of immune cell infiltration and keratinocyte apoptosis, which was not performed due to the patient’s critical condition and high risk of procedure-related complications. Finally, as a single case report, the immunopathological findings and clinical experience cannot be generalized to all patients receiving pucotenlimab, and require further validation in larger multicenter cohorts.

## Conclusion

In this case, pucotenlimab was temporally associated with and considered the most likely trigger of fatal TEN after rigorous exclusion of alternative causes, including β-lactam antibiotics and thoracic radiotherapy. The combination of low PD-1 expression on circulating T cells and markedly elevated IL-6 levels provides exploratory evidence supporting a mechanism of dysregulated T-cell activation and systemic inflammatory response.

For patients receiving PD-1 inhibitors, close clinical monitoring for early mucocutaneous manifestations, timely recognition of suspicious cutaneous lesions, and immediate multidisciplinary intervention are essential to prevent progression to life-threatening severe cutaneous adverse events. Further studies are required to validate these findings and to better define predictive biomarkers for severe immune-related adverse events. This case provides important post-marketing safety data for pucotenlimab and highlights the need for increased vigilance for severe cutaneous irAEs even after a single dose of PD-1 inhibitors.

## Data Availability

The raw data supporting the conclusions of this article will be made available by the authors, without undue reservation.
